# The structural maintenance of chromosomes 5 is a possible biomarker for individualized treatment of colorectal cancer

**DOI:** 10.1002/cam4.5074

**Published:** 2022-07-27

**Authors:** Xiaoxia Gong, Xiaowei Tian, Hao Xie, Zhaoshui Li

**Affiliations:** ^1^ School of Life Science and Technology, MOE Key Laboratory of Developmental Genes and Human Diseases Southeast University Nanjing China; ^2^ General Surgery Department Qingdao Municipal Hospital affiliated to Qingdao University Qingdao China; ^3^ Qingdao Medical College Qingdao University Qingdao China

**Keywords:** biomarker, oxaliplatin, raltitrexed, SMC5, tumor suppressor

## Abstract

**Background:**

Although the understanding of resistance to oxaliplatin (OXA) chemotherapy in colorectal cancer (CRC) has been sought for many years, drug tolerance remains a major challenge for cancer therapy. Revealing the molecular mechanism of OXA resistance could help to explain the poor prognosis of patients.

**Methods:**

Gene expression omnibus (GEO) database was searched, GSE83129, which contains RNA profiling in metastatic CRC patients treated first‐line with OXA, was chosen for the following analysis. Differential expressed genes (DEGs) between the adenocarcinoma and adjacent_normal team, respectively, in the OXA responders and no‐responders were analyzed. The Gene Ontology (GO) and hub genes in the protein–protein interaction (PPI) network were used for the molecular mechanism of OXA resistance. Tumor‐related databases were used for the clinical relevance of the structural maintenance of chromosomes 5 (*SMC5*) in CRC. The in vitro assays were used to detect the molecular function of *SMC5* in CRC cells. Quantitative real‐time PCR (qRT‐PCR) and western blot were used to detect the expression of the structural maintenance of chromosomes 5/6 (SMC5/6) complex components upon OXA and raltitrexed (RTX) treatment. CCK‐8 was used to detect the cell viability of cells with different treatment.

**Results:**

*SMC5* was downregulated in CRC tissues of OXA no‐response patients. Lower expression of *SMC5* was correlated with a poor prognosis in CRC patients, improved this gene expression, inhibited the CRC cell growth and invasion in vitro. Furthermore, *SMC5* was downregulated upon OXA treatment in CRC cells, while RTX would reverse its expression, and the combination of these two drugs restored the SMC5 level to the normal situation. Finally, RTX treatment enhanced the OXA cytotoxicity.

**Conclusion:**

*SMC5* is a tumor suppressor, that low expression of this gene is benefit for the development of CRC. Combination treatment with RTX and OXA may be more suitable for those OXA no‐responders with lower *SMC5*.

## Graphical abstract

In CRC patients of OXA_responders, SMC5 is expressed normally, the proliferation and migration of the cancer cells were decreased (Left). In CRC patients of OXA_no‐responders, SMC5 is downregulated in the cancer cells, OXA treatment further decreased it resulting in extremely low SMC5 content, the proliferation and migration of the cancer cells were increased (Middle). In CRC patients of OXA_no‐responders, SMC5 is downregulated in the cancer cells, the combination treatment of OXA and RTX could not decreased SMC5, the proliferation and migration of the cancer cells were decreased (Right).

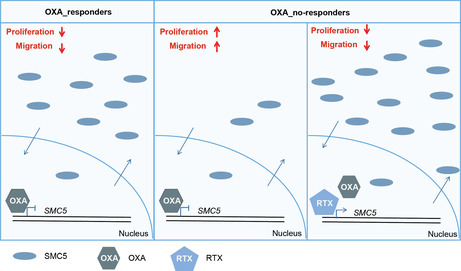

## 1 INTRODUCTION

As the main chemotherapy regimens in colorectal cancer (CRC), a third generated platinum drug oxaliplatin (OXA),^1^, ^2^ to a certain extent, has solved the treatment problem. However, despite advances in the understanding of tumor and the discovery of multiple therapeutic approaches, such as immunotherapy, the development of CRC treatment has been evolutionary but revolutionary. These are resulted from the heterogeneity and evolution of tumor itself.^3^, ^4^, ^5^, ^6^ Genomic instability contributes to the genetic heterogeneity with tumors, providing the genetic diversity required by cancer evolution and enabling the broad phenotypic diversity that is often observed in patients.^7^, ^8^, ^9^ Meanwhile, amplifying genomic instability through chemotherapy has emerged as a powerful but non‐selective of killing cancer cells.^10^, ^11^ Therefore, precision medicine has put forward targeted treatment measures for cancer cells based on their genotype.

Cells have evolved a variety of proteins to ensure the correct replication of DNA, for the orderly division of cells and survival of the organism. The structural maintenance of chromosomes (SMC), which encompasses three classes of structurally and functionally conserved complexes: cohesin, condensin, and SMC5/6 complex, are of the most important protein complexes involved in the genomic stability maintenance.^12^, ^13^, ^14^ Recent cancer studies have indicated that SMC subunits had cancer‐related mutations, for example, cohesin might fall into the most frequently mutated network in cancer.^15^, ^16^ In addition, different deletions of condensin components genomes were found in 12 common cancers.^15^, ^16^ Previous reports have forecasted the cancer‐related mutations in the genome of *SMC5*, an irreplaceable molecular of SMC5/6 complex, which contains a heterodimer composed of SMC5 and SMC6, as well as six non‐SMC elements (NSMCE1_NSMCE4A, SLF1, and SLF2).^17^, ^18^ However, the feasible function of SMC5 in human tumorigenesis is not reported.

To improve the prognosis, combinations of several chemotherapeutic agents, such as combination regimes incorporating irinotecan, OXA, and capecitabine, are now all established options for use as first‐line, second‐line, and sequential treatment of CRC.^19^ In addition, a combination of raltitrexed (RTX) and OXA is verified to have benefit and safety in liver‐only metastatic CRC (mCRC) with chemoresistant disease.^20^ This paper aimed to find new therapeutic targets by understanding the biological mechanisms of OXA resistance. Therefore, here, we found a new biomarker, *SMC5*, to predict the effects of OXA, and to provide options for the combination treatment with RTX of OXA.

## 2 MATERIALS AND METHODS

### 2.1 GEO Data Acquisition and Analysis

The gene expression profiling of GSE83129^21^ was acquired from the National Center for Biotechnology Information (NCBI) Gene Expression Omnibus (GEO) database (https://www.ncbi.nlm.nih.gov/geo/). Differential expressed genes (DEGs) and the following bioinformatics analysis refer to the published literature. The Venn analysis was performed via the Draw Venn Diagram database (http://bioinformatics.psb.ugent.be/webtools/Venn/),^22^ and Gene Ontology (GO)^23^ was analyzed by Metascape (https://metascape.org/gp/index.html#/main/step1).^24^ The protein–protein interaction (PPI) and top 20 hub genes were obtained by STRING database (https://cn.string‐db.org/)^25^ and visualized by Cytoscape software. Gene set enrichment analysis (GSEA)^26^ was analyzed by WebGestalt (http://www.webgestalt.org/).^27^

### 2.2 Tumor‐related database analysis

The individual gene expression level and the prognostic significance of the mRNA expression of *SMC5* were evaluated by the cancer genome atlas (TCGA) database (https://www.cancer.gov/about‐nci/organization/ccg/research/structural‐genomics/tcga).^28^ The overall survival (OS) of CRC patients was analyzed by a Kaplan–Meier survival plot with the bound of the median expression of the gene.

The *SMC5* mRNA expression level based on individual cancer stages was analyzed via UALCAN database (http://ualcan.path.uab.edu/).^29^

The clinical relevance of *SMC5* was analyzed via TIMER database (https://cistrome.shinyapps.io/timer/).^30^

The gene alteration of *SMC5* was evaluated using cBioPortal Cancer Genomics (http://www.cbioportal.org/).

Masked Somatic Mutation data were acquired from Genomic Commons Data Portal GDC (https://xenabrowser.net/) of TCGA as the somatic mutation data in colon adenocarcinoma (COAD) patients. VarScan software was used to preprocess the original data, and maftools R package^31^ was used to visualize the situation of somatic mutation.

### 2.3 Cell culture and treatment

HCT116 and SW480 cell lines were purchased from ATCC. All cell lines were routinely checked for mycoplasma contamination. Cells were grown in DMEM medium with 10% fetal bovine serum (FBS) (#61870–010, Gibco) and 1% penicillin mixed with streptomycin (PS), and the cells were grown at 37°C in a humidified atmosphere of 5% CO_2_ and 95% air.

For OXA treatment, CRC cells were treated with various concentrations of OXA (0 μM, 10 μM, 20 μM, and 40 μM). DMSO without drug served as a control. After 24 h of incubation in cells, cell viability analysis was conducted. For the RTX treatment, CRC cells were treated with various concentrations of RTX (0 nM, 4 nM, 16 nM, 64 nM, 256 nM, and 1024 nM). DMSO without drug served as a negative control. After 24 h and 48 h of incubation, cells were analyzed for cell viability.

### 2.4 RNA extraction, cDNA synthesis, and quantitative real‐time PCR

Total RNA was isolated using RNA isolator (#R401‐01‐AA, Vazyme Biotech) following the manufacturer's protocol. Then, the cDNA library was constructed using HiScript II Q RT SuperMix for qPCR Reverse Transcription Kit (#R223‐01, Vazyme Biotech) according to the manufacturer's protocol. Quantitative real‐time PCR (qRT‐PCR) was performed using AceQ® Universal SYBR® qPCR Master Mix (#Q511, Vazyme Biotech). Primers were synthesized by Integrated DNA Technologies. The relative expression levels of genes were calculated via the 2^−ΔΔCt^ method. The geometric mean of *GAPDH* was used as normalizer for studies. The primers used in qRT‐PCR were listed in Supplementary Table S5.

### 2.5 Plasmid construction and transfection

The mammalian transient expression for CDS of *SMC5* was constructed by cloning the sequences into pEGFP×2‐N1 (#86775, Addgene) vector. The CDS sequence of *SMC5* was achieved with the specific primers. The target cells were transfected with Lipofectamine 2000 (#11668–019, Thermo Fisher) according to the manufacturer's recommendations when reaching 70%–80% confluence.

The cancer cell lines persistently knockdown of *SMC5* using the specific shRNA was constructed referred to the manufacturer's protocol of the vector pLKO.1‐TRC Cloning Vector (#10878, Addgene). A scramble (SHC) sequence was used as a control. The primers used in plasmid construction were listed in Supplementary Table S6.

### 2.6 Whole protein fractionation and western blot

The total protein lysated from whole cell was generated using RIPA (#89900, Thermo Fisher) that contains protease inhibitor (Pierce, #A32963, Thermo Fisher) according to the manufacturer's protocol. Protein concentration was quantified using BCA kit (#23227, Thermo Fisher) following the manufacturer's protocol. In total, 10 μg of proteins were loaded on 8% gels and transferred to nitrocellulose membranes. The corresponding primary antibodies were listed in Supplementary Table **S7**. Immunoreactivity was detected by incubation with ECL (#A38555, Thermo Fisher), and then detected by the autoradiographic film. The gray value was calculated by Image J software.

### 2.7 Cell viability and cell counting

The viability experiments were generated using the cell counting kit‐8 (#HY‐K0301, MCE) according to the manufacturer's instructions. Cells were plated in 96‐well plates with the speeding density of 2 × 10^3^ cells per well, and viability was continuously monitored each day until day 5. The cell viability was calculated relative to day 1.

For the cell counting assay, cells were plated in 24‐well plates at a seeding density of 5 × 10^5^ cells per well, and the dead cells were marked by trypan blue, and the living cells were counted manually and continuously each day until day 5. The cell growth rate was calculated normalized to day 1.

### 2.8 Colony‐forming assays

Cells were plated in 6‐well plates with the speeding density of 500 cells per well and then incubated in fresh media for up to 13 days. Cells were fixed using 4% paraformaldehyde for 15 min at the room temperature, stained with 1% crystal violet, and washed with distilled water. The number of colonies per well was counted manually and calculated using Image J software. Colonies with more than 50 cells were counted.

### 2.9 Data statistics and analysis

Statistical analyses were carried out with GraphPad Prism 7 and SPSS software. The detailed statistical schemes refer to the published articles.

## 3 RESULTS

### 3.1 *SMC5* is decreased in CRC tissues of OXA no‐responders compared to the adjacent normal tissues

**FIGURE 1:**
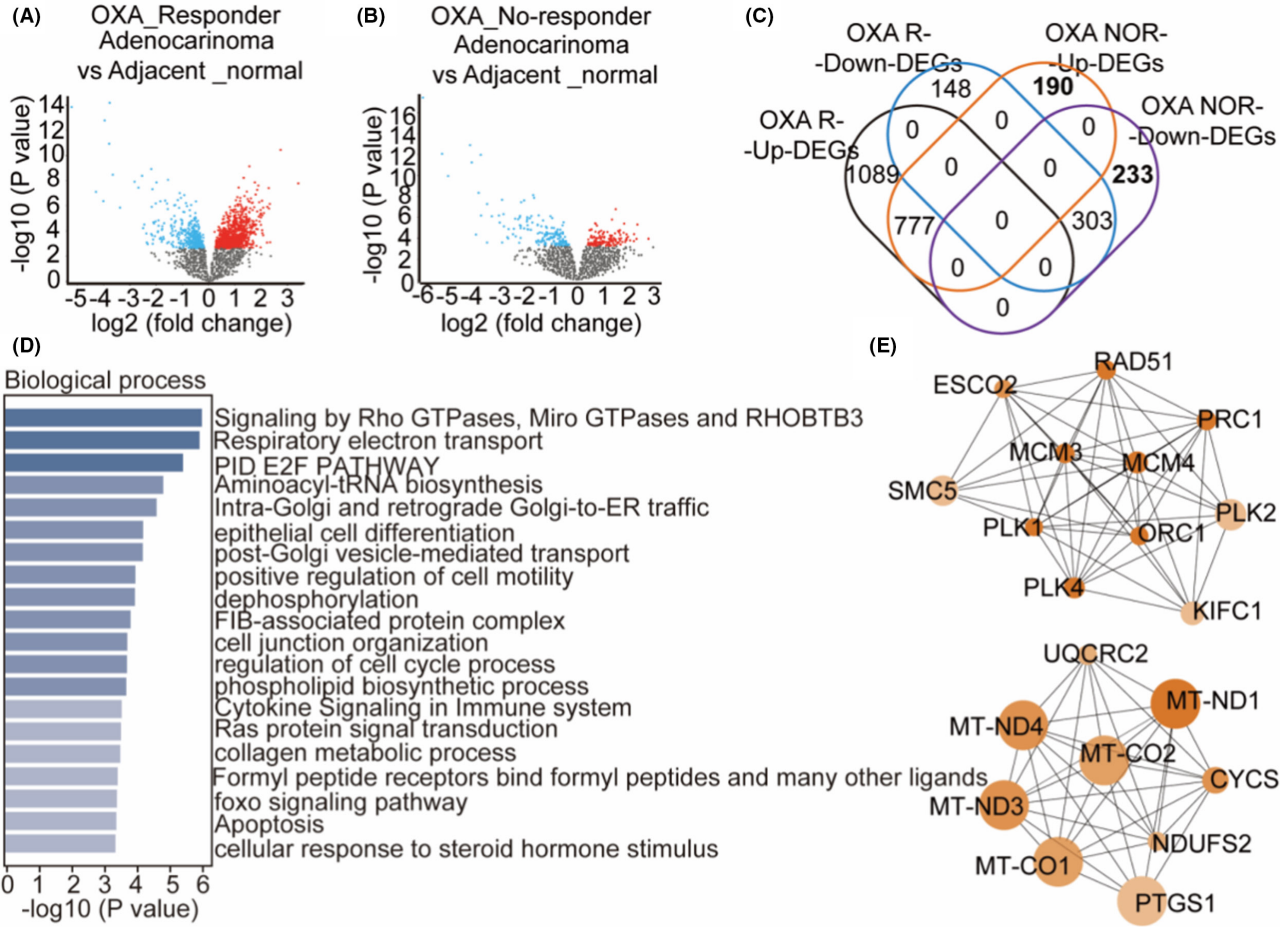
SMC5 is decreased in colorectal cancer tissues of OXA no‐responders compared to control. (A, B) Volcano plots showing the DEGs with the threshold of fold change ≥1.5 and *p* value <0.05 in CRC tissues of OXA_Responder (A) and OXA_No‐responder (B). The blue points represent the downregulated genes, the red points represent the upregulated genes, the gray points represent genes with no significance. (C) Venn drawing showing the 190 upregulated and 233 downregulated genes in CRC tissues of OXA_No‐responder. (D) Enriched GO terms (biological process) of CRC‐related genes in OXA_No‐responder solely. (E) The top 20 hub genes of the 423 DEGs in OXA_No‐responder. OXA R‐Down‐DEGs: OXA_Responder downregulated genes; OXA R‐Up‐DEGs: OXA_Responder upregulated genes; OXA NOR‐Down‐DEGs: OXA_No‐responder downregulated genes; OXA NOR‐Up‐DEGs: OXA_No‐responder upregulated genes.

We searched GEO database and chose GSE83129 (Supplementary Table S1), which contains RNA profiling in metastatic CRC patients treated first‐line with OXA. According to the dataset information, samples were divided into the group that responded to OXA (OXA_Responder) and the group that did not (OXA_No‐responder). Based on the filtering threshold (Fold change: 1.5, *p*‐value<0.05), there were 2317 differential expressed genes (DEGs) (Adenocarcinoma vs Adjacent_normal) in group OXA_Responder (Figure 1A, listed in Supplementary Table S2) and 1505 DEGs (Adenocarcinoma vs Adjacent_normal) in group OXA_No‐responder (Figure 1B, listed in Supplementary Table S3). To analyze DEGs contributed to OXA resistant, 190 upregulated and 233 downregulated DEGs existed in group OXA_No‐responder alone were selected via Venn (Figure 1C, listed in Supplementary Table S4). The subsequent GO analysis showed DEGs existed in group OXA_No‐responder alone belonged to categories that are related to Signaling by Rho GTPases, Miro GTPases, and RHOBTB3, Respiratory electron transport, PID E2F PATHWAY, cell differentiation, cell cycle regulation, and apoptosis (Figure 1D). The top 20 hub genes resulted from PPI network were cell cycle‐related genes (*SMC5*, *ESCO2, RAD51, MCM3, MCM4, PLK1, PLK2, PLK4, ORC1, PRC1, KIFC1*) and respiratory electron transport‐related genes (*UQCRC2, ND1, ND3, ND4, CO1, CO2, CYCS, NDUFS2, PTGS1*) (Figure 1E). The cell cycle‐related genes both in the OXA_Responder and OXA_No‐responder groups were analyzed further. The result showed *SMC5* was significantly downregulated in OXA_No‐responder group while had no change in OXA_Responder (Table 1), On the other hand, *PLK2*, *PLK1*, *PLK4*, *ORC1*, and *KLFC1* were upregulated in OXA_No‐responder group (Table 1).

**TABLE 1:** The relative expression of the cell cycle‐related hub genes respectively in OXP responders and no‐responders

Gene symbol	Fold change		*p*‐value	
	R	NOR	R	NOR
*SMC5*	0.7758	0.4656	0.1890	0.0127
*PLK2*	1.1058	2.6811	0.3400	0.0217
*PLK1*	1.2390	1.5037	0.1280	0.0024
*PLK4*	1.3735	1.7435	0.1600	0.0459
*ORC1*	1.2200	1.5339	0.1350	0.0009
*RAD51*	1.2599	1.5866	0.0258	0.0019
*ESCO2*	1.4670	1.5209	0.0766	0.0484
*MCM3*	1.3828	1.6118	0.0286	0.0059
*MCM4*	1.4895	1.7840	0.0746	0.0055
*PRC1*	1.4608	1.6121	0.0554	0.0536
*LIFC1*	1.5102	1.9236	0.0304	0.0020

Abbreviations: NOR, OXA No‐responder; R, OXA responder.

These results indicated that lower expressed *SMC5* might contribute to OXA resistance of CRC, which has two possible reasons. One is lower expressed *SMC5* might conduce to tumor evolution and malignant metastasis, the other is downregulation of *SMC5* might generate OXA resistance of CRC.

### 3.2 *SMC5* has the feature of tumor suppressor

**FIGURE 2:**
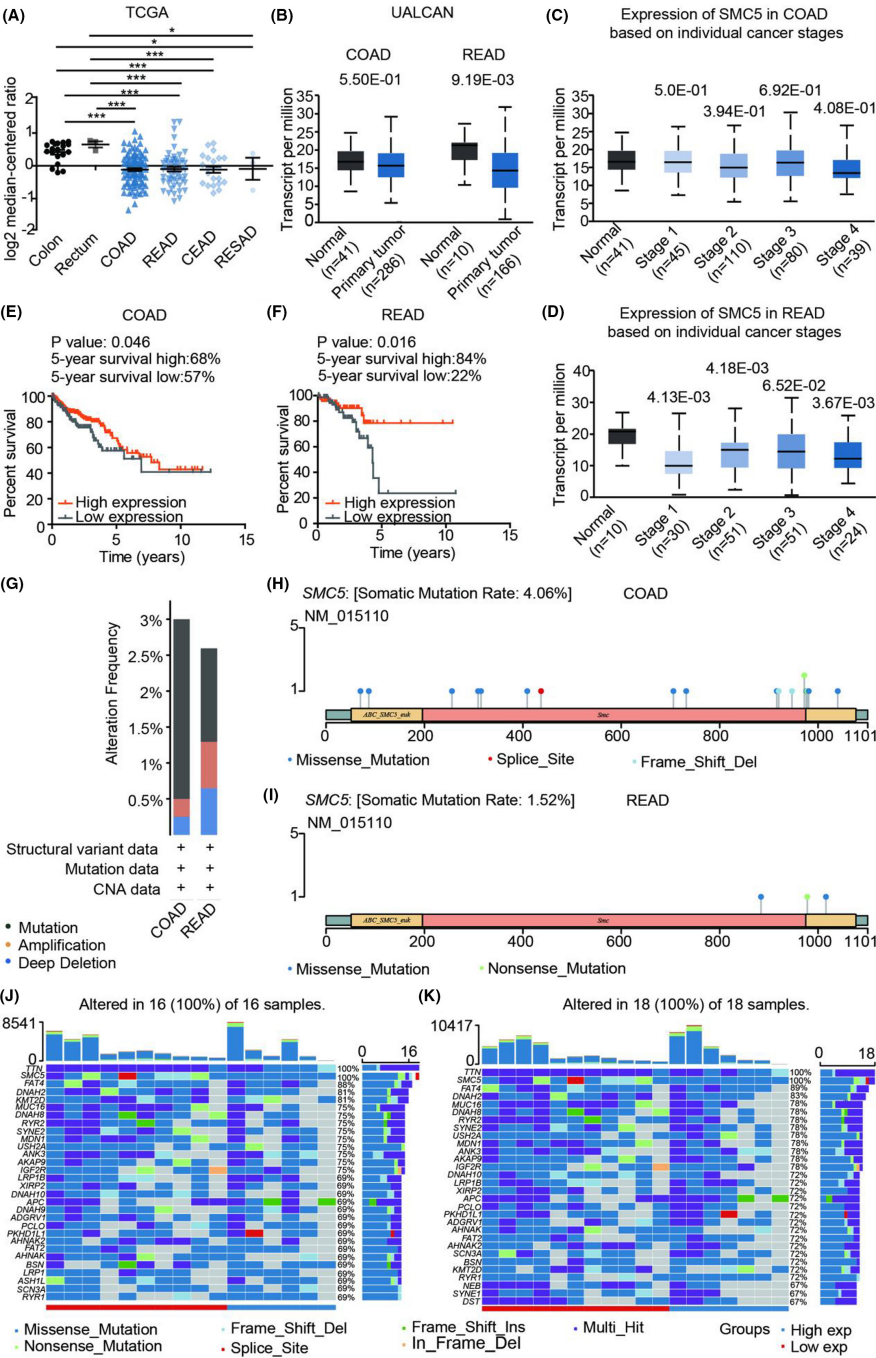
SMC5 is a potential tumor suppressor during CRC tumorigenesis. (A) *SMC5* is decreased in CRC tissues based on sample types in TCGA database. The data statistics was performed via unpaired *t‐test*, ***p < 0.001. (B) *SMC5* is decreased in CRC tissues based on sample types in UALCAN database. (C, D) *SMC5* is decreased in CRC tissues based on individual cancer stages in UALCAN database. E, F The Kaplan–Meier curve showing the relationship of *SMC5* and the prognosis in COAD (E) and READ (F). The average number of log2 median‐centered ratio in all patients was chosen as the boundary of high expression and low expression. The data statistics was performed via Log‐rank (Mantel‐Cox) Test. (G) The alteration frequency of *SMC5* in CRC cells. H, I The mutation site of *SMC5* in COAD (H) and READ (I). (J, K) Top 30 mutant genes in *SMC5* mutation samples of COAD (J) and READ (K). COAD: colon adenocarcinoma; READ: rectal adenocarcinoma.

Then, we analyzed *SMC5* expressional level in CRC samples in TCGA and UALCAN database. The mRNA level of *SMC5* was significantly downregulated in CRC (colon adenocarcinoma (COAD), rectal adenocarcinoma (READ), cecum adenocarcinoma (CEAD), and restosigmoid adenocarcinoma (RESAD)) compared to the normal tissues (colon and rectum) based on sample types (Figure 2A,B) and individual cancer stages (Figure 2C,D). In addition, the Kaplan–Meier curve showed *SMC5* was associated with a good prognosis of the tumor (COAD and READ) (Figure 2E,F), which suggested that lower expressed *SMC5* was more beneficial for the tumor survival. Furthermore, *SMC5* had a significant relevance to the clinical indication of age and stage (Table 2).

**TABLE 2:** The clinical relevance of SMC5 in COAD and READ

	coef	HR	95% CI_I	95% CI_u	*p*‐value	Sig.
COAD						
Age	0.036	1.036	1.012	1.061	0.003	**
Gender						
Male	0.195	1.215	0.701	2.106	0.488	
Race						
Black	−0.630	0.533	0.063	4.500	0.563	
White	−0.705	0.494	0.061	3.997	0.509	
Stage						
Stage 2	0.275	1.316	0.415	4.170	0.641	
Stage 3	0.947	2.579	0.803	8.279	0.111	
Stage 4	2.052	7.785	2.318	26.144	0.001	**
Purity	−0.368	0.692	0.146	3.268	0.642	
Read						
Age	0.126	1.14E+00	1.029	1.25E+00	0.011	*
Gender						
Male	0.417	1.52E+00	0.205	1.22E+01	0.683	
Race						
Black	17.178	2.89E+00	0.000	Inf	0.999	
White	15.700	6.56E+06	0.000	Inf	0.999	
Stage						
Stage 2	−0.931	3.94E‐01	0.044	3.98E+00	0.403	
Stage 3	−0.472	6.24E‐01	0.089	4.37E+00	0.635	
Stage 4	−1.641	1.94E‐01	0.016	2.30E+00	0.194	
Purity	2.307	1.00E+01	0.000	3.70E+03	0.444	

Abbreviations: COAD, colon adenocarcinoma; READ, rectal adenocarcinoma.

*p < 0.05.

**p < 0.01.

cBioPortal for Cancer Genomic database was also investigated and *SMC5* showed a 3% and 2.6% alteration frequency, respectively, in COAD and READ (Figure 2G). Among these, mutations within the functional domain of the protein accounted for the highest proportion (Figure 2G‐I), indicating an indispensable role of *SMC5* in CRC tumorigenesis. In addition, most of the mutations in CRC occur in *SMC5* altered group (Figure S1A–D), which indicated that *SMC5* and other genes have mutation crossover in CRC. Actually, tumor common mutated genes, like *TTN*, *FAT4*, and *DNAH2*, were highly altered in *SMC5* mutation samples, respectively, with the alteration frequency of 100%, 88%, and 81% in COAD (Figure 2J), and 100%, 89%, and 83% in READ (Figure 2K). However, the alteration frequency of *SMC5* has no significant effect on the prognosis of in CRC (Figure S1E,F). These all demonstrated that *SMC5* has the feature of tumor suppressor, that its disorder might contribute to the tumorigenesis and progression of CRC.

### 3.3 Ectopic overexpression of *SMC5* has an inhibitory role in CRC growth in vitro

**FIGURE 3:**
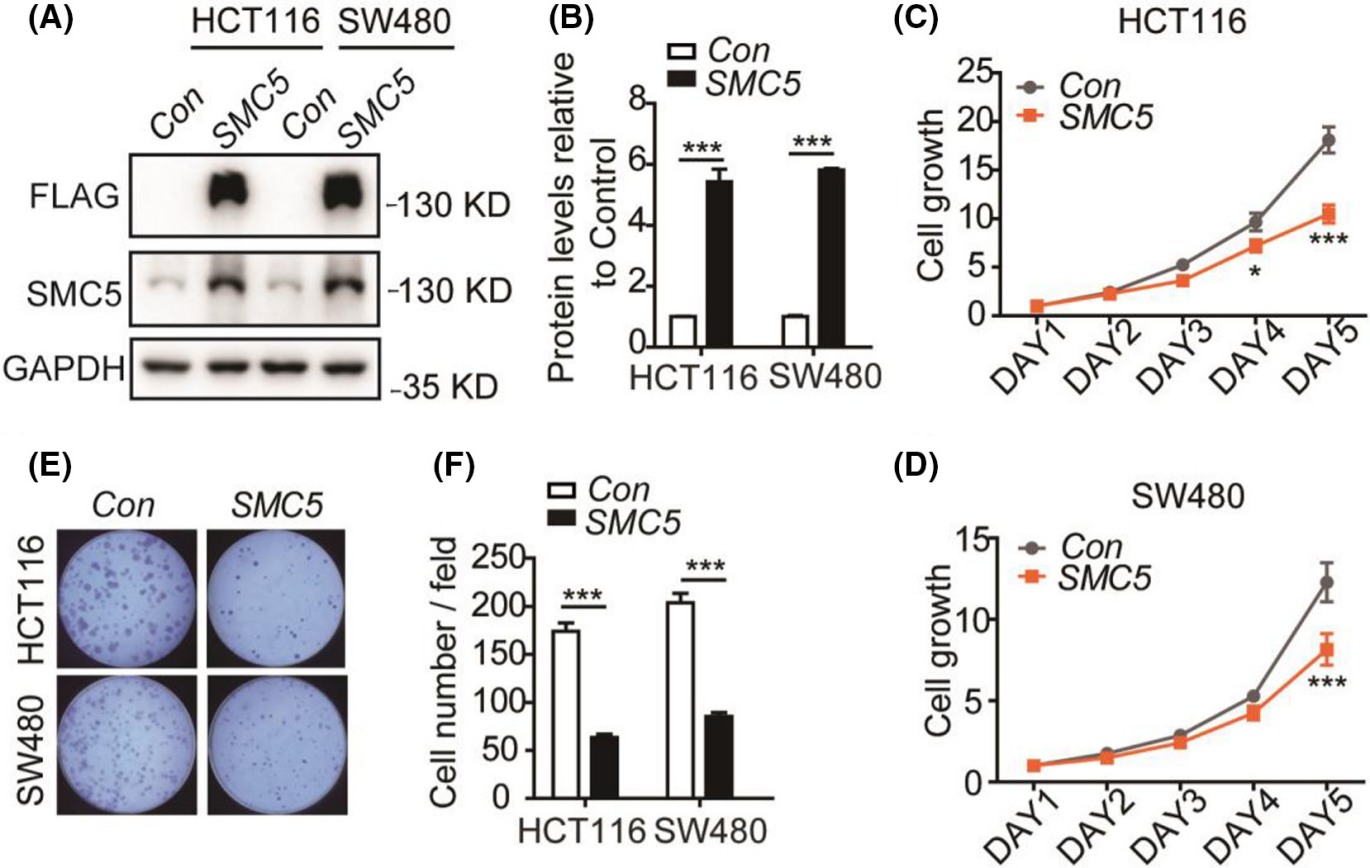
Ectopic overexpression of *SMC5* has an inhibitory role in CRC growth. (A) Intracellular validation of the ectopic overexpressed plasmid. (B) The histogram showing the statistical results of gray values in Figure A. The statistical significance from at least three independent repeats was calculated via one‐way ANOVA. ***p < 0.001. (C, D) Cell counting showing the cell proliferation curve of *SMC5*‐overepressed HCT116 (C) and SW480 (D). The data statistics was calculated via two‐way *ANOVA*. ***p < 0.001. (E) Colony formation of *SMC5*‐overexpressed HCT116 and SW480 cells. The data statistics was calculated via two‐way *ANOVA*. ***p < 0.001.

To assess the molecular function of *SMC5*, human *SMC5* was overexpressed by transfection with a plasmid carrying the *SMC5*‐CDS sequence as well as the 3 × FLAG tag in HCT116 and SW480 cells (Figure 3A,B). The growth level of CRC cells (HCT116, SW480) with *SMC5* overexpression was detected subsequently, which showed that ectopic overexpression of *SMC5* had inhibitory effect on CRC cell growth in vitro, especially as the time goes on (Figure 3C,D). Moreover, the clonality of CRC cell with elevated *SMC5* expression was restrained (Figure 3E,F), suggesting that SMC5 level was critical for the growth of CRC cells in in vitro.

### 3.4 OXA treatment decreases SMC5/6 components levels, while RTX restores them

**FIGURE 4:**
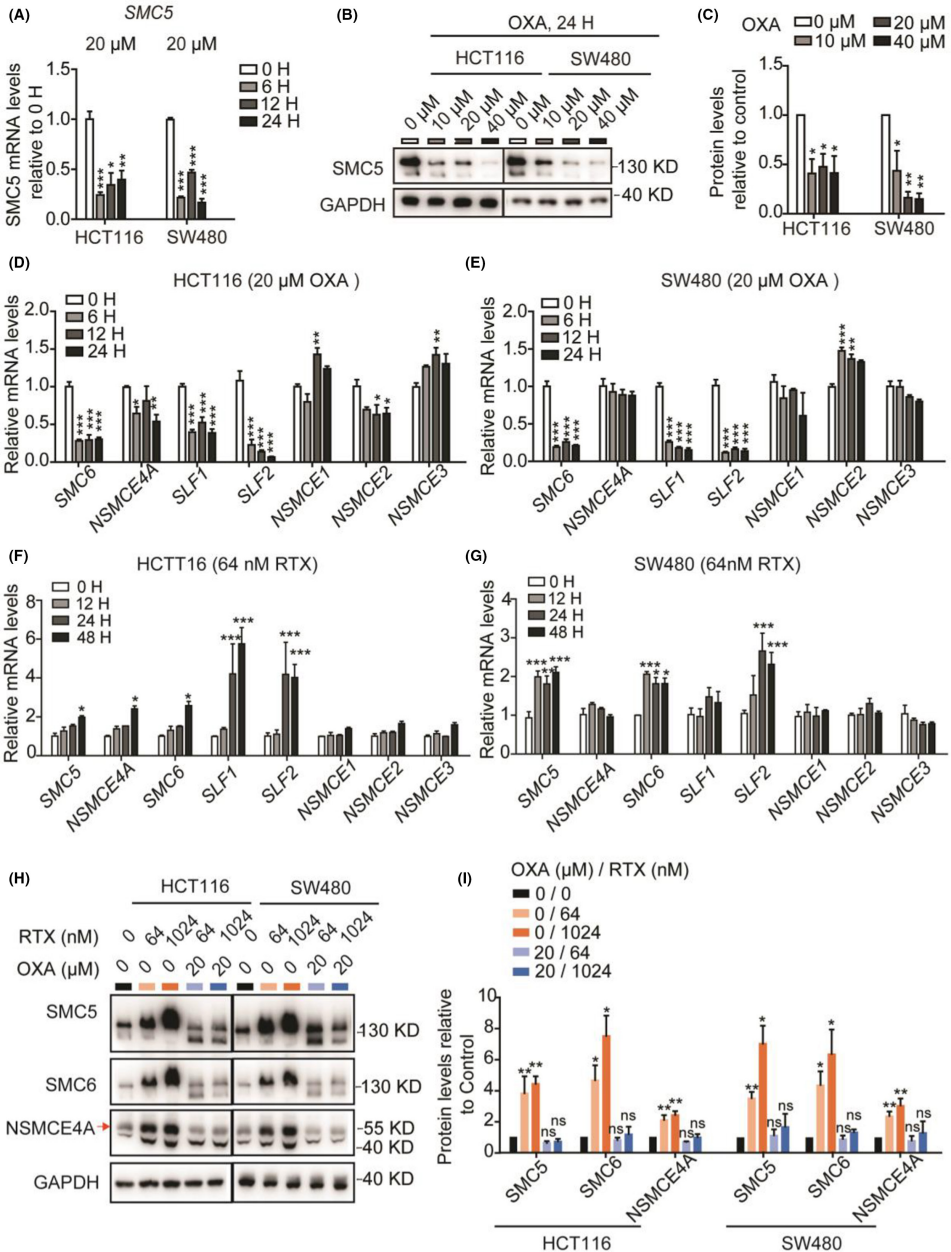
OXA treatment decreases SMC5/6 component levels, while RTX restore them. (A) qRT‐PCR results showing the relative mRNA level of *SMC5* in CRC cells treated by 20 μM OXA for different times. The data statistics was calculated via two‐way *ANOVA*. ***p < 0.001. (B) Western blot results showing the protein levels of SMC5 in CRC cells treated by OXA with different concentration for 24 h. The data statistics from at least three independent repeats was performed via one‐way *ANOVA*. ***p < 0.001. (C) The histogram showing the statistical results of gray values in Figure B. The statistical significance from at least three independent repeats was calculated via one‐way ANOVA. **p < 0.01, *p < 0.05. D, E qRT‐PCR results showing the relative mRNA level of other SMC5/6 components in HCT116 (D) and SW480 (E) treated by 20 μM OXA for different times. The data statistics from at least three independent repeats was calculated via two‐way *ANOVA*. ***p < 0.001, **p < 0.01, *p < 0.05. (F, G) qRT‐PCR showing the expression of SMC5/6 components in HCT116 (F) and SW480 (G) treated by 64 nM RTX for different times. The data statistics from at least three independent repeats was calculated via two‐way *ANOVA*. ***p < 0.001, **p < 0.01, *p < 0.05. (H) Western blot shows the protein levels of SMC5, SMC6, and NSMCE4A in CRC cells with different treatment. (I) The histogram showing the statistical results of gray values in Figure H. The data statistics from at least three independent repeats was performed via one‐way *ANOVA*. **p < 0.01, *p < 0.05, ^ns^ *P*: no significance.

We then detected *SMC5* expressional level upon OXA treatment with an appropriate dose (Figure S2A,B). Both mRNA and protein level of SMC5 was dramatically downregulated (Figure 4A–C), which suggested that the downregulation of SMC5 might be benefit for the cancer cells and may therefore result in a poor prognosis of patients. It is well known that SMC5 is an important backbone protein of the SMC5/6 complex. Hence, both SMC6 and NSMCE4A expression levels were also downregulated upon *SMC5* knockdown (Figure S2A,B), indicating that the overall stability of the complex may be reduced by SMC5 impairment. Furthermore, in OXA‐treated CRC cells, the mRNA levels of *SMC6*, *NSMCE4A*, *SLF1*, and *SLF2* changed similarly to *SMC5* (Figure 4D,E), while *NSMCE1*, *NSMCE2*, and *NSMCE3* changed insignificantly (Figure 4D,E), suggesting that the effect of SMC5 on CRC cells may be mediated through the whole SMC5/6 complex. This was consistent with the correlation analysis on expression level of the SMC5/6 components in COAD and READ (Figure S4A–N). We noticed that the mRNA level of *NSMCE4A* was not changed significantly in SW480 upon OXA treatment. We consider that this may be due to the different sensitivity of *NSMCE4A* to OXA in different cell. We also found that *NSMCE1_3* had a different trend from *SMC5* in OXA‐treated CRC cells. However, although the protein levels of NSMCE4A and SMC6 were decreased in both CRC cells with *SMC5* knockdown (Figure S3A,B), the mRNA level of these genes was not affected (Figure S3C–E). These all indicated that the transcriptional of the complex genes may not be uniformly regulated, but the proteins would be affected together. The reduction in one protein leads directly to the complex disorder.

Chemotherapy combination is usually used to solve the single drug resistance problem in clinical practice. We searched GEO database and analyzed the expression levels of SMC5/6 in CRC cell lines treated with different chemotherapeutic agents, including doxorubicin (GSE116441), gemcitabine (GSE116444), lapatinib (GSE116445), sorafenib (GSE116448), and topotecan (GSE116450).^32^ However, we did not find the increased tendency on the expression levels of SMC5/6 upon these treatments. We then detected the effect of RTX on SMC5/6 components in CRC cells, with an appropriate concentration (Figure S5A,B). Both mRNA and protein level of SMC5/6 components (*SMC5*, *SMC6*, *NSMCE4A*, *SLF1*, and *SLF2*) were upregulated significantly upon RTX treatment (Figure 4F–I). In addition, combination treatment of OXA and RTX could restore the expression level of SMC5/6 components to the normal level compared to the OXA‐only treatment (Figure 4H,I). These results indicated that the combination treatment of RTX and OXA might enhance the therapeutic effect for the patients with low SMC5.

### 3.5 RTX enhances the OXA cytotoxicity of CRC cells

**FIGURE 5:**
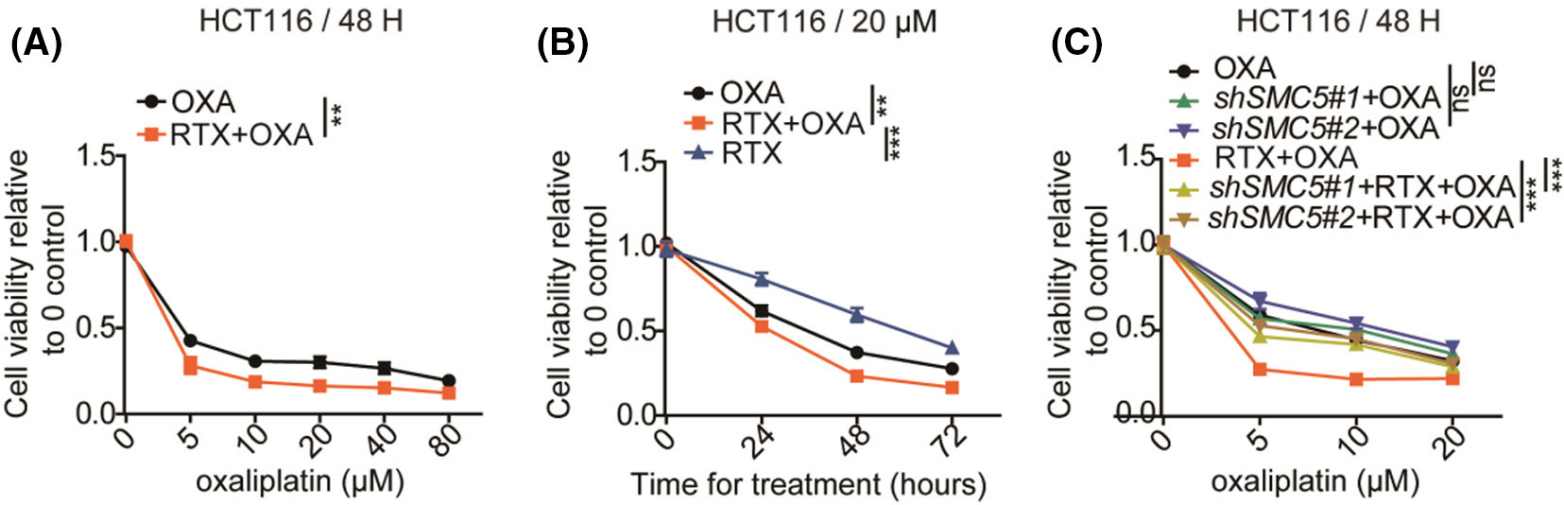
Raltitrexed enhances the OXA cytotoxicity of CRC cells. (A) CCK‐8 results showing the cell viability of HCT116 following OXA, or the combined treatment with RTX (64 nM), and the different concentration (0 μM, 5 μM, 10 μM, 20 μM, 40 μM, 80 μM) of OXA for 48 h, relative to control (treated with no drug). The data statistics from at least three independent repeats was conducted by two‐way *ANOVA*. ***p < 0.001, **p < 0.01. (B) CCK‐8 results showing the cell viability of HCT116 following OXA (20 μM), RTX (64 nM), or the combined treatment, for 0, 24, 48, and 72 hours, relative to control (treated with no drug). The statistical significance from at least three independent repeats was calculated via two‐way *ANOVA*. ***p < 0.001, **p < 0.01. (C) CCK‐8 results showing the cell viability of HCT116 with *SMC5* knockdown or not respectively following OXA or the combined treatment of OXA (0 μM, 5 μM, 10 μM, 20 μM) and RTX (64 nM) for 48 h relative to control (treated with no drug). The data statistics from at least three independent repeats was performed via two‐way *ANOVA*. ***p < 0.001. OXA, oxaliplatin; RTX, raltitrexed; OXA + RTX: the combined treatment of OXA and RTX.

To further demonstrate the toxicity effect of the combination treatment of RTX and OXA, the cell viability of cells was detected. As expected, the combination of RTX enhanced cell susceptibility to OXA (Figure 5A,B). These indicated that the combination is more lethal than the two drugs alone. Meanwhile, blocked expression of *SMC5* significantly decreased the drug susceptibility of HCT116 to the combined treatment (Figure 5C), while had no effect on OXA cytotoxicity (Figure 5C), indicating the important role of SMC5 in the combined treatment. These all suggested that in those patients, who are not respond to OXA due to lower *SMC5* level, combination treatment of RTX may be a better choice.

## 4 DISCUSSION

As the third most life‐threatening disease in the world, CRC is characterized by genomic instability.^33^, ^34^ There are many clinical treatments for CRC, and the most common is a combination of the primary therapy with surgery and adjuvant therapy with chemotherapy, which usually is the combination of platinum (OXA, cis‐platinum, and lobaplatin) and fluorouracil (5‐FU) or RTX.^35^

Our results demonstrate a potential tumor suppressor *SMC5* was significantly downregulated in CRC tissues of OXA no‐response patients, which suggested that lower expression of SMC5 may contribute to OXA resistance. *SMC5* is essential for the accurate sister chromatid separation during cell division. Cells lacking *SMC5* exhibit structural malformation in their chromosomes and the further genome instability,^36^, ^37^, ^38^ which could be the initial cause of tumorigenesis or the malignant metastasis.^39^, ^40^, ^41^ Our further analysis showed that lower expression of *SMC5* had a poor prognosis in CRC patients, while improved SMC5 expression had a significant inhibitory effect on the cancer cell growth.

Meanwhile, our analysis verifies that SMC5 was downregulated upon OXA treatment, suggesting that for those patients with lower SMC5, OXA therapy may further reduce SMC5 level, which may further contribute to the malignant development of cancer cells. Therefore, the use of OXA alone should not be recommended for patients with low expression of SMC5. In this case, RTX may be a better choice or a candidate for combination with OXA, since RTX could significantly upregulate the expression level of SMC5, and the combination with OXA could restore the level of SMC5 to the normal situation. Most of all, the combined treatment also has the strongest cytotoxicity compared with the OXA or the RTX treatment alone, and this might depend on SMC5. This may provide a certain theoretical basis for individualized treatment of different groups of clinical colon cancer.

As the skeleton protein of SMC5/6 complex, SMC5 functions in multiple fields, such as DNA repair,^41^, ^42^ viral genome transcriptional inhibition,^43^, ^44^ regulation of meiosis and mitosis,^45^, ^46^ rDNA replication, and telomere stability maintenance.^47^, ^48^ All these functions are usually dependent on the complex. In this paper, we also figured out the tumor suppressor function of SMC5 was inextricably linked to the SMC5/6 complex, with the evidence that other components of SMC5/6 complex were also changed after OXA and/or RTX treatment, and these changes followed the same trend as those of SMC5. These indicate that the whole SMC5/6 complex has a tumor suppressive function, and the specific molecular biological mechanisms involved remain to be further investigated.

Besides *SMC5*, 422 genes were also differentially expressed in the population of OXA_No‐responder alone. Changes in the expression of these genes may also contribute significantly to OXA resistance, especially for the hub genes. The top 20 genes are enriched in two different biological processes: cell cycle regulation and respiratory electron transport. Dysregulation of cell cycle machinery always causes genome instability and is associated with chemoresistance in CRC. For example, upregulated PLK1 signaling correlates with the poor prognosis in CRC patients, and blockade of it increases the OXA sensitivity.^49^ Higher expression level of PLK2 significantly predicted a poorer outcome in patients with CRC. Knockdown of these genes leads to the enhanced cellular apoptosis induced by OXA, and elevated expression of it enhances the resistance of CRC cells to chemotherapeutic agents.^50^ Additionally, respiratory electron transport‐related genes were also reported to be linked to the prognosis and chemotherapeutics resistance. For instance, as an important subunit of mitochondrial respiratory complex III, ubiquinol‐cytochrome c reductase complex core protein 2 (UQCRC2) is reported to play an important role in the tumorigenesis and progression of CRC and revealed to be a novel prognostic and therapeutic target.^51^ Although the specific molecular mechanism of these related genes was not illuminated here, our results on the hand further confirmed these conclusions, and on the other hand, suggested further work is needed to analyze other cell cycle regulated genes as their potential roles in the chemotherapeutics resistance.

However, several limitations of this present study should be stated. First, despite the fact that gene expression changes can be analyzed using GEO database, this analysis was only performed on gene mRNA expression levels. Therefore, clinical CRC samples treated with OXA should be collected to further detect the protein level of SMC5. Second, OXA‐resistant cell lines should be further used to analyze the impact of SMC5 expression levels on OXA resistance. Finally, the in vivo experiments also should be performed to further determine the function of SMC5 in OXA resistance. Moreover, this potential biological function of SMC5 may depend on the integrity of the SMC5/6, thus, whether the function of OXA resistance is related to the genome instability is also the direction of further research.

Altogether, here, we demonstrate that SMC5 is a possible novel biomarker for individualized treatment of CRC. For those OXA no‐responders with low SMC5, RTX treatment may be the best option for subsequent treatment.

## AUTHOR CONTRIBUTIONS

Conceptualization, Xiao GONG; Data curation, Xiao GONG and Hao Xie; Formal analysis, Xiao GONG; Funding acquisition, Hao Xie; Investigation, Xiao TIAN and Zhao LI; Methodology, Xiao TIAN and Zhao LI; Project administration, Hao Xie; Supervision, Hao Xie; Validation, Hao Xie; Visualization, Xiao GONG; Writing – original draft, Xiao GONG; Writing – review & editing, Hao Xie.

## CONFLICT OF INTEREST

The authors report no conflicts of interest for this work.

## ETHICS STATEMENT

Ethical approval was not required for this review article.

## Supporting information


Figure S1‐S5

Table S1

Table S2

Table S3

Table S4

Table S5

Table S6

Table S7

## Data Availability

Not application.
