# Supplementary material for: The structural maintenance of chromosomes 5 is a possible biomarker for individualized treatment of colorectal cancer
Source: Cancer Med. 2022 Jul 27;12(3):3276–87. doi: 10.1002/cam4.5074 (PMC9939147; doi:10.1002/cam4.5074)
Supplement: Supplementary file 1 — Figure S1‐S5 [file CAM4-12-3276-s007.docx]

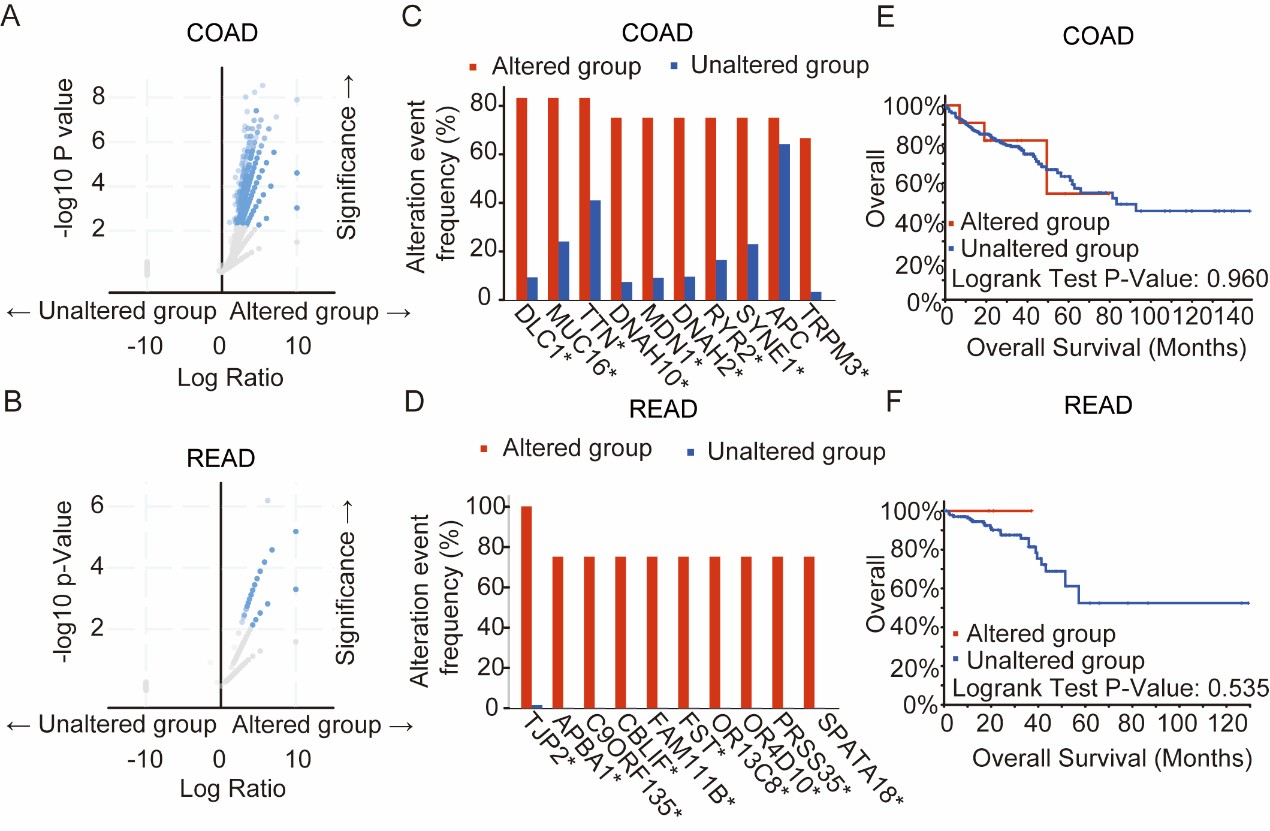


**Supplementary Figure 1 Analysis of *SMC5* alteration frequency in CRC. A-D** The mutation frequency of other genes in SMC5 altered groups and unaltered groups. **E, F** The relationship of SMC5 alteration frequency with CRC prognosis.


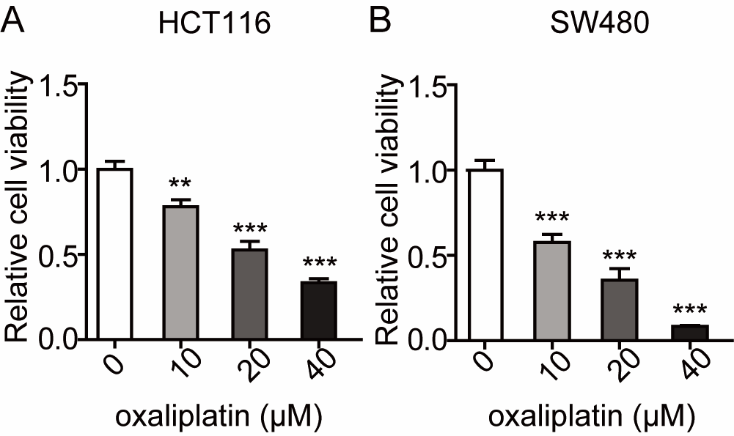


**Supplementary Figure 2 The cell viability of CRC cells treated with appropriate concentration of OXA. A, B** The relative cell viability of HCT116 (A) and SW480 (B) upon OXA treatment with different concentration to the control cell (0 μM). The statistical significance from at least three independent repeats was calculated via student’s *t-test*. ^***^*P*<0.001, ^**^*P*<0.01.


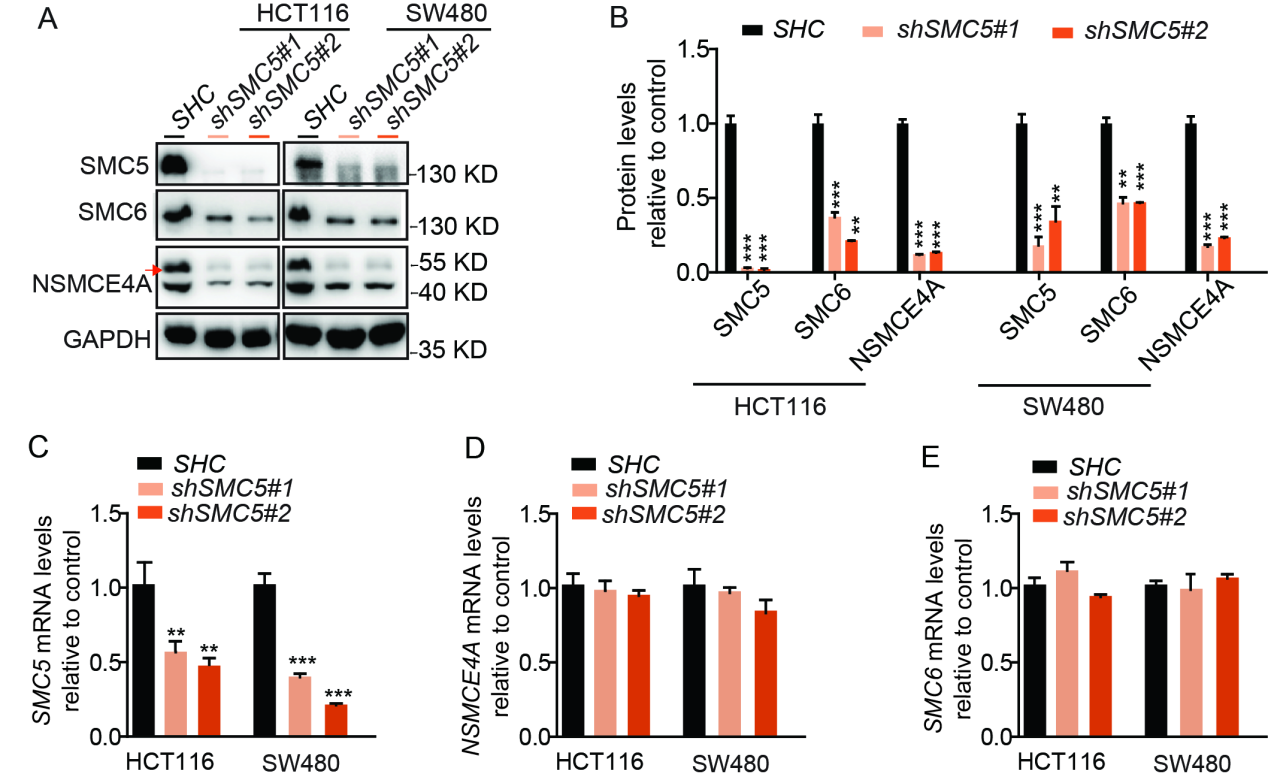


**Supplementary Figure 3 SMC6 and NSMCE4A are down-regulated by SMC5 knockdown.** **A** Western blot showing the proteins levels of SMC5, NSMCE4A, and SMC6 in CRC cells with *SMC5* knockdown. B The statistical results of the gray value of figure A. The statistical significance from at least three independent repeats was calculated via one-way *ANOVA*. ^***^*P*<0.001, ^**^*P*<0.01. **C-E** qRT-PCR showing the mRNA levels of *SMC5* (C), *NSMCE4A* (D), and *SMC6* (E) in CRC cells with *SMC5* knockdown. The statistical significance from at least three independent repeats was calculated via one-way *ANOVA*. ^***^*P*<0.001, ^**^*P*<0.01.


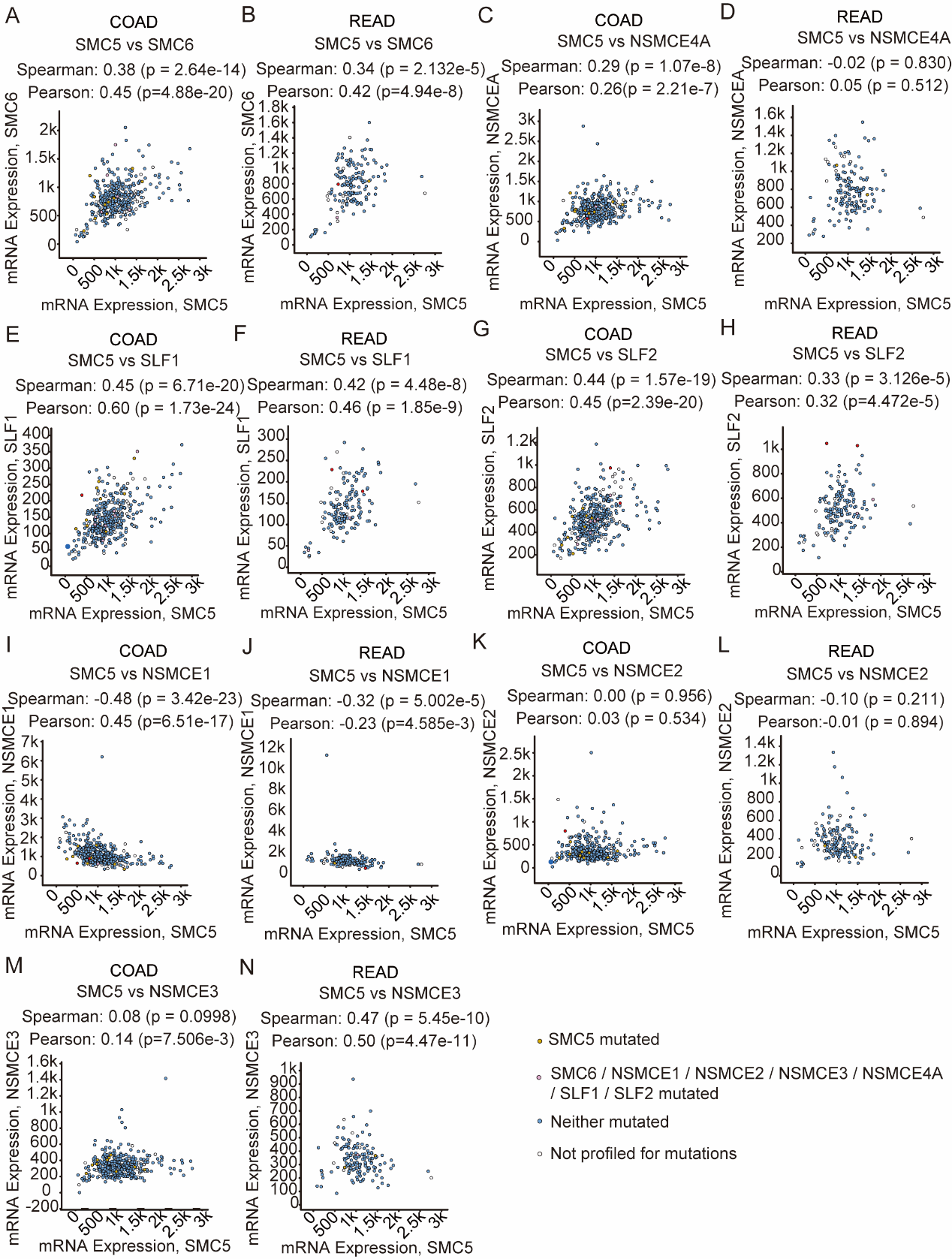


**Supplementary Figure 4 The co-expression correlation of SMC5 and other SMC5/6 components in colon adenocarcinoma.** **A-N** Data were searched in the public database cBioPartal for Cancer Genomics.


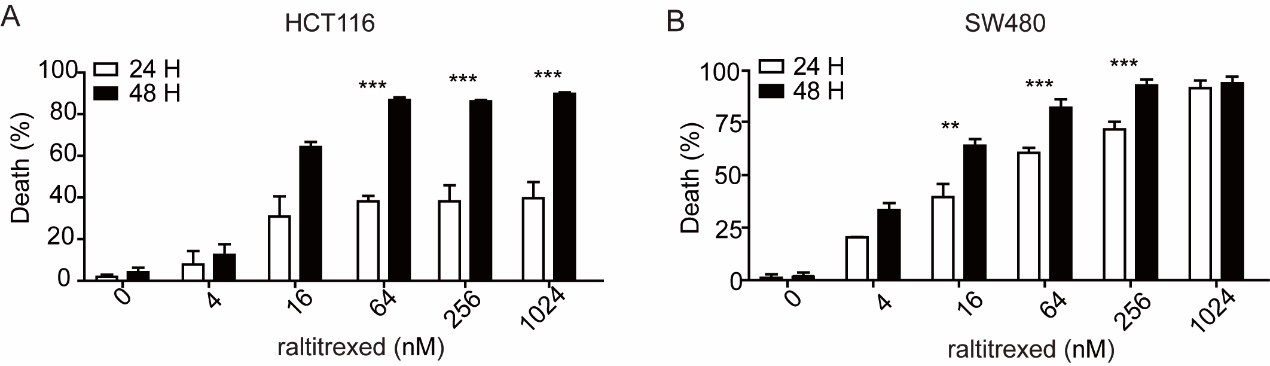


**Supplementary Figure 5 The cell death of CRC cells treated with appropriate concentration of raltitrexed. A, B** The percentage of cell death of HCT116 (A) and SW480 (B) upon raltitrexed treatment with different concentration. The statistical significance from at least three independent repeats was calculated via two-way *ANOVA*. ^***^*P*<0.001, ^**^*P*<0.01, ^*^*P*<0.05.
